# Unveiling a Tunable Moiré Bandgap in Bilayer Graphene/hBN Device by Angle‐Resolved Photoemission Spectroscopy

**DOI:** 10.1002/advs.202412609

**Published:** 2025-01-23

**Authors:** Hanbo Xiao, Han Gao, Min Li, Fanqiang Chen, Qiao Li, Yiwei Li, Can Li, Meixiao Wang, Fangyuan Zhu, Lexian Yang, Shiyong Wang, Feng Miao, Yulin Chen, Cheng Chen, Bin Cheng, Jianpeng Liu, Zhongkai Liu

**Affiliations:** ^1^ School of Physical Science and Technology, ShanghaiTech Laboratory for Topological Physics ShanghaiTech University Shanghai 201210 P. R. China; ^2^ Nanjing National Laboratory of Solid State Microstructures, School of Physics, Institute of Brain‐Inspired Intelligence, Collaborative Innovation Center of Advanced Microstructures Nanjing University Nanjing 210093 P. R. China; ^3^ Institute for Advanced Studies Wuhan University Wuhan Hubei 430072 P. R. China; ^4^ Key Laboratory of Artificial Structures and Quantum Control (Ministry of Education), School of Physics and Astronomy Shanghai Jiao Tong University Shanghai 200240 P. R. China; ^5^ Center for Transformative Science ShanghaiTech University Shanghai 201210 P. R. China; ^6^ Shanghai Synchrotron Radiation Facility, Shanghai Advanced Research Institute Chinese Academy of Sciences Shanghai 201204 P. R. China; ^7^ State Key Laboratory of Low Dimensional Quantum Physics, Department of Physics Tsinghua University Beijing 100084 P. R. China; ^8^ Department of Physics, Clarendon Laboratory University of Oxford Parks Road Oxford OX1 3PU UK; ^9^ Institute of Interdisciplinary Physical Sciences School of Science Nanjing University of Science and Technology Nanjing 210094 P. R. China

**Keywords:** moiré potential, bilayer graphene, nanoARPES with gating, bandstructure engineering

## Abstract

Over the years, great efforts have been devoted in introducing a sizable and tunable band gap in graphene for its potential application in next‐generation electronic devices. The primary challenge in modulating this gap has been the absence of a direct method for observing changes of the band gap in momentum space. In this study, advanced spatial‐ and angle‐resolved photoemission spectroscopy technique is employed to directly visualize the gap formation in bilayer graphene, modulated by both displacement fields and moiré potentials. The application of displacement field via in situ electrostatic gating introduces a sizable and tunable electronic bandgap, proportional to the field strength up to 100 meV. Meanwhile, the moiré potential, induced by aligning the underlying hexagonal boron nitride substrate, extends the bandgap by ≈20 meV. Theoretical calculations effectively capture the experimental observations. This investigation provides a quantitative understanding of how these two mechanisms collaboratively modulate the band gap in bilayer graphene, offering valuable guidance for the design of graphene‐based electronic devices.

## Graphical abstract

Direct visualization of moiré band structure and tunable bandgap formation in hBN‐aligned bilayer graphene is achieved using advanced NanoARPES with in situ electrostatic gating. This study reveals how displacement fields and moiré potentials collaboratively modulate electronic properties, providing new insights into bandgap engineering for next‐generation graphene‐based electronic devices.

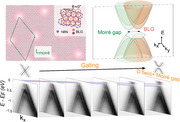

## 1 Introduction

The band gap in a semiconductor is one of the crucial factors that determines many electrical and optical properties of the system and key to the device performance, for instance, the on/off ratio of a field‐effect transistor and the absorption wavelength of a photodetector. In recent years, there has been a substantial interest in the research of 2D materials due to their versatile physical properties.^[^ ^1^, ^2^, ^3^, ^4^ ^]^ Specifically, these materials exhibit bandgaps spanning from terahertz/mid‐infrared to the visible and ultraviolet regions,^[^ ^5^, ^6^, ^7^ ^]^ making them promising candidates for various device applications.

Graphene stands out as a leading 2D material for electronic applications due to its unique properties, including unparalleled electrical and thermal conductivities, high electron mobility, and remarkable mechanical flexibility.^[^ ^8^, ^9^ ^]^ However, the absence of a bandgap in the intrinsic graphene system severely limits its potential applications.^[^ ^10^ ^]^ To create a sizable and controllable bandgap, great efforts have been devoted such as electrostatic gating,^[^ ^5^, ^11^, ^12^, ^13^, ^14^, ^15^ ^]^ atom intercalation,^[^ ^16^ ^]^ substrate doping,^[^ ^17^ ^]^ size confinement,^[^ ^18^ ^]^ rhombohedral stacking,^[^ ^19^ ^]^ and the introduction of moiré potentials.^[^ ^1^, ^7^, ^20^, ^21^, ^22^, ^23^, ^24^, ^25^, ^26^, ^27^, ^28^, ^29^, ^30^, ^31^ ^]^ These bandgap tuning mechanisms could effectively modulate the gap size and tune the performance of graphene‐based devices.

Understanding the interplay of these tuning methods requires a detailed investigation of their impact on graphene's physical properties, including changes in lattice structure, impurity levels, and electronic band structure. Although various characterization techniques (mostly indirect) including transport measurements,^[^ ^2^, ^6^, ^12^, ^32^, ^33^, ^34^, ^35^, ^36^ ^]^ scanning tunneling microscopy,^[^ ^15^ ^]^ and optical methods^[^ ^5^, ^37^, ^38^, ^39^, ^40^ ^]^ etc., have provided insights into the effects of these tuning methods, a comprehensive and direct observation of the momentum‐space evolution of the band structure under multiple tuning knobs remains elusive. Such direct visualization is crucial for unraveling the underlying principles of the bandgap engineering in graphene, which could provide essential insights into the development of graphene‐based electronic devices.

In this study, exploiting state‐of‐the‐art spatial‐ and angle‐resolved photoemission spectroscopy (NanoARPES) with in‐situ electrostatic gating, we systematically investigate the electronic structure and the evolution of the bandgap in a Bernel‐stacked bilayer graphene (BLG) device, in the presence of both moiré potential (through the moiré superlattice formed by hexagonal boron nitride (hBN) substrate aligned with graphene layers) and displacement field (D‐field, via electrostatic gating).

By comparing the result with BLG devices without moiré superlattice (by forming large twist angle between BLG and hBN), we find that while the band gaps in both BLG devices could be tuned continuously by the D‐field up to 100 meV, the moiré potential could further enhance the band gap size by ≈20 meV, which is consistent with our theoretical calculations. These findings provide a direct momentum‐space visualization of the corroborative modulations of the gap size in bilayer graphene tuned by both D‐field and moiré potential. Specifically, our results emphasize the significance of the moiré potential as an effective tuning mechanism for inducing substantial band gaps in graphene systems, and its potential in engineering the electronic structure of other applicable 2D devices.

## 2 Results

**Figure 1:**
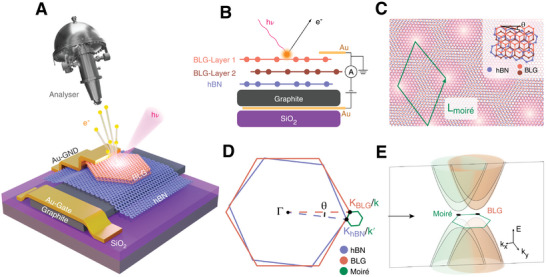
NanoARPES on BLG/hBN heterostructure. A) Schematics of the experimental setup. B) Side‐view of the sample and gating geometries. C) Real‐space illustration of BLG lattice with almost aligned hBN substrate with a twist angle of *θ*. The generated moiré unit cell is indicated by the green rhombus. D) Illustration of the Brillouin zones (BZs) of hBN (blue), BLG (orange). The mini‐BZ generated from the moiré lattice is labelled in green. *K*_BLG_/ *K*_hBN_ and *k/k’* label the high symmetry points in the extended BZ and the mini‐BZ, respectively. E) Schematic showing the replication of Dirac‐shape main bands by the moiré potential in the mini‐BZ.

We conducted NanoARPES measurements to explore the electronic structure of both BLG devices with and without moiré superlattice. The experiment setup is illustrated in **Figure** **1**A (3D schematic) and 1B (schematic side view of the sample). The BLG device is grounded through the top Au contact, and a graphite/Au electrode is placed at the bottom of the hBN to serve as a back gate, which enables the electrostatic tuning of both the D‐field and electron filling in BLG. The moiré potential in BLG is introduced by intentionally aligning the underlying hBN substrate to the graphene layers (with a twisted angle of 0.3°, see details in Methods). The small lattice mismatch between graphene and hBN enables the formation of a moiré lattice with a real‐space periodicity (*L*_moiré_) around 12 nm (Figure 1C). Correspondingly, the moiré lattice generates mini‐Brillouin zones (mini‐BZs) around K point of BLG with a reciprocal vector (*G*_moiré_) of 0.053 Å^−1^ (Figure 1D), which replicates the Dirac‐shape band dispersion of BLG into wider momentum space (illustrated in Figure 1E).

**Figure 2:**
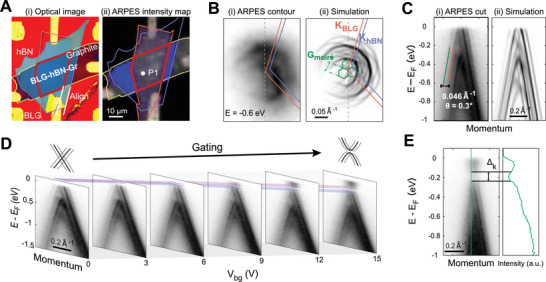
Electronic structure of BLG with moiré superlattice and its evolution with in situ electrostatic gating. A) Real space information of the BLG/hBN sample with moiré superlattice. (i) Optical image and (ii) NanoARPES real space intensity map. Data presented in B–E) was taken at P1. B) Constant energy contours near the *K*_BLG_ at *E* = *E_F_* −0.6 eV for (i) ARPES and (ii) simulation.^[^ ^41^ ^]^ BZ of BLG, hBN and mini‐BZ are labelled. C) Band dispersion across *K*_BLG_ along the direction indicated by the grey dashed line in (B), for (i) ARPES and (ii) simulation. The momentum distance between BLG band and its replica (orange and green line) is measured to be 0.046 Å^−1^, corresponding to a twist angle of 0.3° between hBN and BLG. D) Evolution of the band dispersion with consecutive back gate voltages. The color stripes indicate the evolution of the CNP and opening of the bandgap. E) Band dispersion cut taken at *V_bg_* = 15 V and the energy distributions curve taken at around K point (green line), where the band gap ∆*_k_* is extracted.

**Figure 3:**
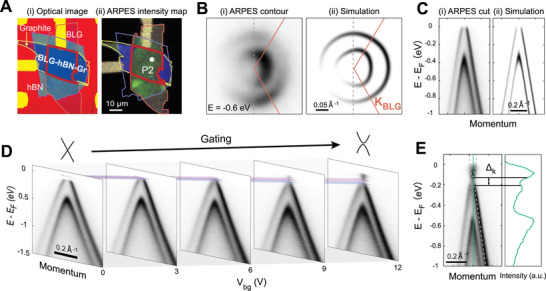
Electronic structure of BLG/hBN without moiré superlattice and its evolution with in‐situ electrostatic gating. A) Real space information of the BLG/hBN sample without moiré superlattice. (i) Optical image and (ii) NanoARPES real space intensity map. ARPES measured at point P2. The boundary of each segment is indicated with the same color in Figure 1. B) Constant energy contours near the *K*_BLG_ at *E* = *E_F_* − 0.6 eV of (i) ARPES and (ii) simulation.^[^ ^41^ ^]^ BZ of BLG is labelled. C) Band dispersion across *K*_BLG_ along the direction labelled by the grey dashed line in B), by (i) ARPES and (ii) simulation. D) Evolution of the band dispersion across *K*_BLG_ along the direction labelled by the grey dashed line in (C) at different back gate voltage. The color stripes indicate the evolution of the CNP and opening of the bandgap. E) Band dispersion across *K*_BLG_ along the direction labelled by the grey dashed line in B) with *V_bg_* = 12 V (left panel) and the plot of the energy distributions curve at around K point (right panel). ∆*_k_* labels the band gap between the valence and conduction band.

In **Figure** **2**, we present the NanoARPES result performed on BLG device with moiré superlattice. The optical microscope image and the real‐space intensity map of NanoARPES are presented in Figure 2A, respectively, where the BLG/hBN regions can be precisely located. A representative constant energy contour taken at −0.6 eV below Fermi level (*E_F_*) is illustrated in Figure 2B(i) (complete electronic structure can be found in Section S1, Supporting Information), where the main band structure of BLG and its moiré replicas are observed. This result is well reproduced by our simulation, considering both the effect of moiré potential and photoemission matrix element^[^ ^41^, ^42^ ^]^ on the BLG band structure (Figure 2B(ii), details see Section S2, Supporting Information). The slight hole doping of the graphene system is likely induced by the influence from hBN substrate. The effect of moiré potential on the Dirac‐shape main bands is more prominently seen in the band dispersion cuts (Figure 2C for both ARPES and simulation), where the moiré replicas are observed on both sides, in sharp contrast with the situation without the moiré superlattice (presented later in **Figure** **3**). The spacing between the main band and its moiré replica is measured to be 0.046 Å^−1^, consistent with the twist angle of 0.3°. The charge neutral point (CNP) is found to locate slightly higher than *E_F_*, thus only accessible with electrostatic gating.

The in‐situ electrostatic gating with positive back gate voltage (*V_bg_*) is then applied on the device, which effectively introduces more electrons into the system and enables the visualization of conduction band. Consecutive dispersion cuts taken with increasing *V_bg_* is presented in Figure 2D, illustrating the evolution of the band structure. Besides the rigid shift of chemical potential, the presence of D‐field brings in energy drop across the two layers of the BLG system, and a band gap at CNP is introduced consequently, whose size monotonically increases with *V_bg_*, in line with previous reports.^[^ ^14^ ^]^ A representative dispersion cut (taken at *V_bg_* = 15V) is shown in Figure 2E, where the size of the band gap is extracted to be around 100 meV (labeled by ∆*_k_*). Notably, the moiré replicas of the main bands are persistent in the presence of D‐field, and no signature of additional in‐gap state is observed (see further analysis in Section S3, Supporting Information).

To better understand the effect of moiré potential on BLG system, we conducted comparative NanoARPES measurement on BLG device without moiré superlattice, and the result is presented in Figure 3. The twist angle between BLG and hBN is examined to be around 15° (see Section S4, Supporting Information for details) in this device, corresponding to a moiré periodicity of 0.95 nm in real space. In this situation, the effect of moiré lattice on the band structure is considered minimum, due to the large moiré vector in momentum space (*G* = 0.66 Å^−1^), thus weak Fourier component of the moiré potential, as compared to the dispersive Dirac‐shape band of graphene in the vicinity of K point. Indeed, the constant energy contour taken at −0.6 eV below *E_F_* (Figure 3B for both ARPES and simulation, complete electronic structure is presented in Section S1, Supporting Information) shows characteristic feature of intrinsic BLG with two concentric rings, deriving from the two Dirac‐shape bands of BLG with an energy difference around 400 meV (Figure 3C for both ARPES and simulation). No signature of moiré modulation on the electronic structure, as found in Figure 2, is evidenced here. Comparatively, we conduct the in‐situ electrostatic gating on this device (Figure 3D), and it shows similar behavior including both the shift of chemical potential and the opening of the band gap (Figure 3E).

**Figure 4:**
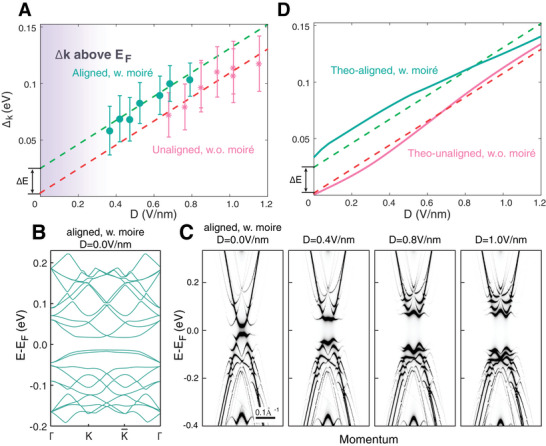
Calculated spectral function and bandgap evolution as a function of D field. A) Plot of the bandgap ∆*_k_* extracted from NanoARPES spectra for BLG with (green) and without (pink) moiré superlattice as a function of D field. The dashed lines indicate the trend of the bandgap evolution, and the energy difference is labeled by ∆*E* ≈ 20 meV. B) Calculated band structures of BLG with moiré superlattice with *θ* = 0° based on tight‐binding model. The high symmetry points are within the moiré mini‐BZ. C) Simulated band dispersion cut along the same direction as Figure 2c with D field for *D* = 0, 0.4, 0.8 and 1.0 V nm^−1^, respectively. D) Plot of the bandgap ∆*_k_* extracted from theoretical calculation for BLG with (green) and without (pink) moiré superlattice as a function of D field. The dashed lines mark the trend of the bandgap evolution taken from experiments as in A).

To quantitatively distinguish the effects of moiré potential and D‐field in BLG system, we extract the sizes of the band gap in each ARPES spectrum and plot them as a function of the D‐field *D = ϵV_bg_/2d* (**Figure** **4**A, here *ϵ* is the dielectric constant and *d* is the thickness of hBN, see Section S5, Supporting Information for details). The evolution of the band gap in BLG devices with and without moiré superlattice shows a similar trend, increasing monotonically with the enhancement of D‐field. Notably, the size of the band gap in BLG system with moiré superlattice is found to be larger than the without case by Δ*E* ≈ 20 meV with the same D‐field, indicating the additional enhancement of band gap from the moiré potential.

To capture these experimental findings in BLG/hBN system, theoretical calculation is performed based on the tight‐binding model (see Method for details). As shown in Figure 4B, the simulated band structure of BLG with moiré superlattice suggests that the moiré potential can sufficiently introduce a band gap ≈ 20 meV at the CNP, due to the C_2z_ symmetry breaking. This is notably different from the monolayer situation, where the hBN substrate can only open the band gap by 2 meV.^[^ ^43^ ^]^ In addition, the band gap in BLG with moiré superlattice could be further enhanced by D‐field, as it increases the difference of the interlayer potential in BLG system. By introducing the D‐field in the model (see Method for details), the simulated spectrum (Figure 4C) well captures the experimental result, in which the band gap increases with the D‐field while the main band and its moiré replicas remain stable. The theoretical results are summarized in Figure 4D (data without moiré superlattice is reproduced from previous reports^[^ ^4^, ^5^ ^]^, and in general captures our experimental findings. We note a kink feature (change of linear slope) at D ≈0.5 V nm^−1^ is predicted by the calculation due to the mutual interaction between the gap generated from both primary and secondary Dirac point as a function of D‐field,^[^ ^44^ ^]^ which is not prominent in our ARPES result (see detailed analysis in Sections S6, S7, Supporting Information).

## 3 Discussion

Our measurement of the moiré band gap (≈20 meV) in the BLG with moiré superlattice is consistent with previous observations using other techniques such as STM (≈9 meV),^[^ ^45^ ^]^ optical measurement (38 meV)^[^ ^46^ ^]^ and photocurrent spectroscopy (≈14  meV).^[^ ^25^ ^]^ The slight difference among these measurements may result from the spatial variation of twist angles and/or local strains^[^ ^47^, ^48^, ^49^ ^]^ in the different devices. It is worth noting that in our ARPES result, the difference of the bandgap size between the samples with and without moiré superlattice remains constant with increasing D‐field, which implies that the overall bandgap can be interpreted as the arithmetic sum of the contributions from the moiré potential and the D‐field induced gaps.

Therefore, the overall bandgap in our bilayer graphene (BLG) system thus can be conceptualized as the cumulative effect of two distinct mechanisms: interlayer potential drop and intralayer C_2z_ symmetry breaking. The D‐field applied across the BLG establishes differential potential energies across its two layers, while alignment with hBN substrate breaks the C_2z_ rotational symmetry. The interaction between graphene and the aligned hBN is well characterized by the moiré potential, which gives rise to secondary moiré replica bands. Together, these mechanisms cooperatively influence the bandgap in the BLG system with moiré superlattice from alignment with hBN, resulting in the gate tunable modulation of its electronic properties.

Furthermore, the observed combined‐effect of the moiré potential and D‐field may help to understand the unique transport properties at the hBN/BLG interface, for example, the ratcheting mechanism for carrier injection in hBN/BLG based moiré synaptic transistor devices.^[^ ^50^ ^]^ Meanwhile, it is proposed that the moiré potential may generate “localized” carriers point at the aligned hBN/BLG interface.^[^ ^51^ ^]^ Our measurement on the hBN‐aligned BLG (with moiré superlattice) provides momentum‐space spectroscopic evidence about the moiré bands whose dispersions remain intact across the large range of the D‐field, which helps reveal the nature of the interface states.

In summary, using state‐of‐the‐art NanoARPES system equipped with in‐situ electrostatic gating, we systematically investigated the evolution of electronic structure of BLG systems with and without moiré superlattice due to alignment/unalignment with hBN substrate. The moiré potential modulated electronic structure of BLG is directly revealed, with the presence of an energy gap at CNP of ≈20 meV, resulting from the C_2z_ symmetry breaking induced by moiré potential. The presence of D‐field can further increase this band gap with a rate of dΔ*_k_*/d*D* ≈0.11 eV per V nm^−1^, due to enhancement of interlayer potential energy difference. Our measurement provides direct spectroscopic evidence of bandgap engineering under multiple tuning knobs in BLG/hBN systems and addresses the importance of moiré potential in reshaping the electronic structure of the system.

These effective tuning results further suggest that in terms of device application, the BLG/hBN heterostructure with moiré superlattice has great advantage over mechanically exfoliated BLG/SiO_2_^[^ ^5^ ^]^ and epitaxial BLG,^[^ ^26^ ^]^ as it exhibits high quality interface, very high mobility,^[^ ^52^ ^]^ and suppressed charge inhomogeneities.^[^ ^53^ ^]^ This modification can be further refined through the application of external fields, strain, or substrate interactions, thereby enhancing the design and functionality of graphene‐based electronic devices.

## 4 Experimental Section

### Device Fabrication

The hBN‐aligned bilayer graphene was fabricated by a modified polymer‐based pick‐up technique. Graphene and hBN flakes were exfoliated on separate SiO_2_/Si substrates, with the thickness of these flakes identified with optical contrast and atomic force microscope (Bruker MultiMode 8). hBN thickness by AFM was measured, the thickness of hBN was 5.9 nm for unaligned sample (twist angle = 15°) and 19.0 nm for aligned one (twist angle = 0.3°), detailed data shown in Section S8 (Supporting Information). A polycarbonate–poly(dimethylsiloxane) (PC/PDMS) stamp was used to pick the bilayer graphene (BLG), hBN and graphite flakes at 80°C sequentially. Then the stack was released on the SiO_2_/Si substrate at 140°C. The polycarbonate film was dissolved by chloroform. The angle between BLG and hBN was measured by AFM and further checked by ARPES. The contact electrodes (Cr 5 nm/Pd 15 nm/Au 40 nm) to BLG and bottom graphite gates were deposited by standard electron‐beam evaporation after patterned by electron beam lithography. The spatial homogeneity of moiré lattice and moiré induced gap addressed by AFM and ARPES presented in Section S9 (Supporting Information).

### NanoARPES Measurement with In Situ Electrostatic Gating

The NanoARPES measurements were performed at the BL07U endstation of Shanghai Synchrotron Radiation Facility (SSRF). The base pressure was lower than 1×10^−10^ mbar. The sample was annealed at 300 °C for 3 hours prior to the measurement to desorb absorbates. The beam was focused using a Fresnel zone plate (FZP) and a spatial resolution of ≈400 nm was achieved. All the experiments were performed at T = 20 K with a photon energy of 92.6 eV and right‐handed circular polarized light with a fixed incidence angle of 60°. The data were collected by a hemispherical Scienta DA30 electron analyzer. The instrumental energy and momentum resolution under the measurement conditions was ≈20 meV and 0.2 Å^−1^, respectively.

### Atomistic Tight‐Binding Model

A realistic atomic tight‐binding model was used to study the electronic structure of bilayer graphene/hBN. In the atomistic tight‐binding model, an empirical Slater Koster‐type parameter *t*(***d***) was used to describe the hopping between the *p_z_* atomic orbitals at different sites^[^ ^54^ ^]^

(1) $$t\;\;\;\left(d\right)=V_{\sigma }^{0}\;e^{-\frac{\left(r-d_{c}\right)}{\delta_{0}}}{\left(\frac{d\cdot \hat{z}}{d}\right)}^{2}+V_{\pi }^{0}e^{-\frac{\left(r-a_{0}\right)}{\delta_{0}}}\left[1-{\left(\frac{d\cdot \hat{z}}{d}\right)}^{2}\right]$$

where $V_{\sigma }^{0}=0.48eV,V_{\pi }^{0}=-2.7eV$, and *d_c_* was 3.35 Å representing the interlayer distance of graphene. *δ_0_* = 0.184*a*, where a was the lattice constant of graphene and $a_{0}=a/\sqrt{3}$. *d* was the displacement vector between two sites. The on‐site potential *V_C_* = 0 for carbon atom was assumed, and *V_B_* = 3.34 eV, *V_N_* = −1.40 eV for boron and nitride atoms, respectively. The unrelaxed condition was used since minimal structural relaxation effects was found in these samples (see detail in Section S10, Supporting Information). The lattice structure was rationalized to the relative lattice period *a*_hBN_/*a* ≈ 1.018 to 56/55 with assumed perfect alignment *θ* = 0°.

### Spectral Function Calculations

To simulate the ARPES intensity, the unfolded spectral functions were calculated for the bilayer system along *k* path in the atomic BZ near the K valley, and the unfolded spectral function was expressed as^[^ ^55^ ^]^

(2) $$A\;\left(\omega ,k\right)=-\frac{1}{\pi }lm\left[\sum_{l\alpha }\sum_{n}\frac{|\langle l\alpha k|nk\rangle {|}^{2}}{\omega -E_{nk}+i\delta }\right]$$

where *l* refers to the layer index and *α* represents the sublattice index. |*n**k*** 〉 was the Bloch wave function at the ***k*** point of the *n*‐th moiré energy band, and |*l*α ***k*** 〉 denotes the Bloch wave function of the *l*‐th monolayer graphene and *α* sublattice, without the moiré potential effects. The *n*‐th moiré Bloch function at wavevector ***k*** could be expressed as

(3) $$\left|nk\rangle \right.=\sum_{il\alpha }\;C_{il\alpha ,n}\left(k\right)\left|il\alpha k\rangle \right.$$

where *i* stands for the atomic lattice vector index within a moiré primitive cell. The Bloch‐like state |*il*α ***k*** 〉 at wavevector ***k*** could be expressed in the basis of the *p_z_*‐orbital‐like Wannier functions as

(4) $$\;\left|il\alpha k\rangle \right.=\frac{1}{\sqrt{N_{M}}}\;\sum_{R}e^{ik\cdot R}\left|il\alpha R\right.\rangle$$

where ***R*** was the moiré lattice vector. Then, it was obtained:

(5) $$\begin{array}{c} \langle l\alpha k|nk\rangle =\sum_{R^{\prime }i^{\prime }l^{\prime }\alpha^{\prime }}\;\langle l\alpha k|i^{\prime }l^{\prime }\alpha^{\prime }R\rangle \;\langle i^{\prime }l^{\prime }\alpha^{\prime }R|nk\rangle =\frac{1}{\sqrt{N_{0}}}\;\sum_{i}e^{-ik\cdot a_{i}}C_{il\alpha ,n}\left(k\right)\;\;\;\; \end{array}$$

in which ***a*** *_i_* refers to the *i*‐th atomic lattice vector within a moiré primitive cell for the *l*‐th layer, and *N_0_* was the number of atomic unit cells within a moiré primitive cell.

## Conflict of Interest

The authors declare no conflict of interest.

## Supporting information



Supporting Information

## Data Availability

The data that support the findings of this study are available from the corresponding author upon reasonable request.
