# Supplementary material for: Unveiling a Tunable Moiré Bandgap in Bilayer Graphene/hBN Device by Angle‐Resolved Photoemission Spectroscopy
Source: Adv Sci (Weinh). 2025 Jan 23;12(11):2412609. doi: 10.1002/advs.202412609 (PMC11923876; doi:10.1002/advs.202412609)
Supplement: Supplementary file 1 — Supporting Information [file ADVS-12-2412609-s001.docx]

**Supplementary Information for**

**Unveiling a Tunable Moiré Bandgap in Bilayer Graphene/hBN Device by Angle-Resolved Photoemission Spectroscopy**

Hanbo Xiao^1^†, Han Gao^1^†, Min Li^1^†, Fanqiang Chen^2^†, Qiao Li^1^, Yiwei Li^3^, Can Li^4^, Meixiao Wang^1,5^, Fangyuan Zhu^6^, Lexian Yang^7^, Shiyong Wang^4^, Feng Miao^2^, Yulin Chen^1,8^, Cheng Chen^1^*, Bin Cheng^9^*, Jianpeng Liu^1^*, Zhongkai Liu^1^*

^1^School of Physical Science and Technology, ShanghaiTech Laboratory for Topological Physics,

ShanghaiTech University, Shanghai 201210, China.

^2^Nanjing National Laboratory of Solid State Microstructures, School of Physics, Institute of Brain-Inspired Intelligence, Collaborative Innovation Center of Advanced Microstructures, Nanjing University, Nanjing 210093, P. R. China.

^3^Institute for Advanced Studies, Wuhan University, Wuhan, Hubei, 430072, P. R. China.

^4^Key Laboratory of Artificial Structures and Quantum Control (Ministry of Education), School of Physics and Astronomy, Shanghai Jiao Tong University, Shanghai, 200240, China.

^5^Center for Transformative Science, ShanghaiTech University, Shanghai 201210, China.

^6^Shanghai Synchrotron Radiation Facility, Shanghai Advanced Research Institute, Chinese Academy of Sciences, Shanghai 201204, China.

^7^State Key Laboratory of Low Dimensional Quantum Physics, Department of Physics, Tsinghua University, Beijing, 100084, China.

^8^Department of Physics, Clarendon Laboratory, University of Oxford, Parks Road, Oxford OX1 3PU, UK.

^9^Institute of Interdisciplinary Physical Sciences School of Science, Nanjing University of Science and Technology, Nanjing 210094, P. R. China.

^*^Corresponding author. Email: [chencheng1@shanghaitech.edu.cn](mailto:chencheng1@shanghaitech.edu.cn) (C.C.), [bincheng@njust.edu.cn](mailto:bincheng@njust.edu.cn) (B.C.), [liujp@shanghaitech.edu.cn](mailto:liujp@shanghaitech.edu.cn) (J.L.), [liuzhk@shanghaitech.edu.cn](mailto:liuzhk@shanghaitech.edu.cn) (Z.L.)

†These authors contributed equally to this work.

**List of contents:**

[Section S1: Electronic Structure of BLG sample with and without moiré superlattice. 3](#_Toc184676749)

[Section S2: Band structure simulation of the BLG/hBN system 4](#_Toc184676750)

[Section S3: Absence of in-gap state with the presence of D-field. 6](#_Toc184676751)

[Section S4: Determination of twist angles of BLG/ hBN devices. 7](#_Toc184676752)

[Section S5: Calculation of the displacement field. 8](#_Toc184676753)

[Section S6: Analysis of secondary Dirac point (SDP) gap. 9](#_Toc184676754)

[Section S7: Deviation of the ARPES extracted Δ_k_ from the theory predictions. 11](#_Toc184676755)

[Section S8: Determination the thickness of hBN substrate. 12](#_Toc184676756)

[Section S9: Homogeneity of the moiré lattice and moiré induced gap. 13](#_Toc184676757)

[Section S10: Relaxation effects within the moiré lattice. 16](#_Toc184676758)

[Section S11: Band gap size obtained through resistivity measurement. 18](#_Toc184676759)

[Section S12: Exclusion of ferroelectricity in our device. 20](#_Toc184676760)

[Section S13: Extraction of Δ_k_ and the estimation of the measurement error. 21](#_Toc184676761)

[References 25](#_Toc184676762)

Section S1: Electronic Structure of BLG sample with and without moiré superlattice.

In Fig. S1, we presented constant energy contours and band dispersion plots of our photoemission spectra, on BLG sample with and without moiré superlattice, respectively, to give a general overview of their electronic structures.


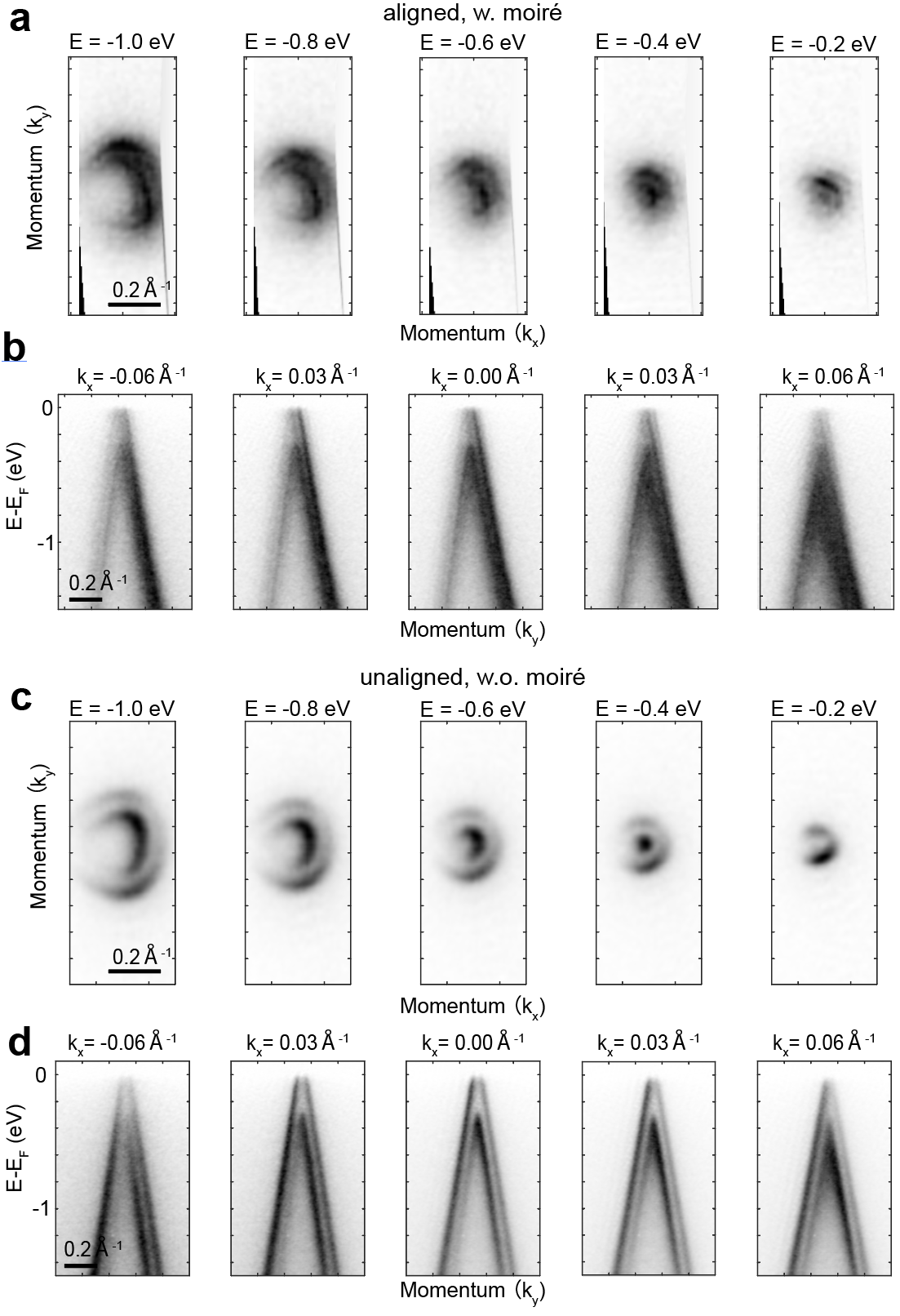


**Fig. S1. Electronic Structure of BLG with and without moiré superlattice sample. a.** Constant energy contours near the *K*_BLG_ at different energy levels of BLG sample with moiré superlattice. **b.** Band dispersion across *K*_BLG_ of BLG sample with moiré superlattice at different *k_x_* momentum along the same direction as Fig. 2C.**c-d**. Same as a-b, but for BLG sample without moiré superlattice, and direction in d the same as Fig. 3C.

Section S2: Band structure simulation of the BLG/hBN system.

In Fig. 2B, C and Fig. 3B, C of the main text, we compared our ARPES result with theoretical simulations, to gain better understanding of the observed band structure. Detailed method for simulation and the extraction of twisted angle are illustrated in the following:

Considering Bloch function of AB stacking BLG $\left| \Phi\right\rangle$ and  $\hat{H}\left| \Phi\right\rangle=E\left| \Phi\right\rangle$. In a primitive cell there are four atoms *A_1_, B_1_, A_2_, B_2_*, $\left| \Phi\right\rangle$ is a linear combination of the periodic wave functions $\left| \Phi\right\rangle=c_{1}\left| A_{1} \right\rangle+c_{2}\left| B_{1} \right\rangle+c_{3}\left| A_{2} \right\rangle+c_{4}\left| B_{2} \right\rangle$. We have an analytical model of the Bernal bilayer graphene including all tight-binding parameters(*1*).

$$\begin{aligned} \left( \begin{matrix} \Delta& \gamma_{0}f & \gamma_{1} & \gamma_{4}f^{*} \\ \gamma_{0}f^{*} & 0 & \gamma_{4}f^{*} & \gamma_{3}f \\ \gamma_{1} & \gamma_{4}f^{*} & \Delta& \gamma_{0}f^{*} \\ \gamma_{4}f & \gamma_{3}f^{*} & \gamma_{0}f & 0 \end{matrix} \right)\left( \begin{matrix} c_{1} \\ c_{2} \\ c_{3} \\ c_{4} \end{matrix} \right)=E\left( \begin{matrix} c_{1} \\ c_{2} \\ c_{3} \\ c_{4} \end{matrix} \right)\#\left( 1 \right) \end{aligned}$$

Where $f\left( \boldsymbol{k} \right)=2\cos\left( \frac{\sqrt{3}b}{2}k_{x} \right)\exp\left( -\frac{ib}{2}k_{y} \right)+\exp\left( ik_{y}b \right)$ is the structure factor. Δ = -0.015 eV, *γ*_0_ = -3.12 eV, *γ*_1_ = 0.38 eV, *γ*_3_ = 0.28 eV, *γ*_4_ = 0.12 eV are four tight-binding parameters.

To reduce the 4×4 matrix of $\hat{H}$, we can introduce a symmetrized basis:

$$\begin{aligned} \left( \begin{matrix} c_{1} \\ c_{2} \\ c_{3} \\ c_{4} \end{matrix} \right)=\left( \begin{matrix} \frac{1}{\sqrt{2}} & 0 & 0 & \frac{1}{\sqrt{2}} \\ 0 & \frac{f^{*}}{\sqrt{2}\left| f \right|} & \frac{f^{*}}{\sqrt{2}\left| f \right|} & 0 \\ \frac{1}{\sqrt{2}} & 0 & 0 & \frac{-1}{\sqrt{2}} \\ 0 & \frac{f^{*}}{\sqrt{2}\left| f \right|} & \frac{-f^{*}}{\sqrt{2}\left| f \right|} & 0 \end{matrix} \right)\left( \begin{matrix} d_{1} \\ d_{2} \\ d_{3} \\ d_{4} \end{matrix} \right)\#\left( 2 \right) \end{aligned}$$

Therefore, the matrix equation $\hat{H}\left| \Phi\right\rangle=E\left| \Phi\right\rangle$ has form:

$$\begin{aligned} \left( \begin{matrix} h_{11} & h_{12} & 0 & 0 \\ h_{21} & h_{22} & h_{23} & 0 \\ 0 & h_{32} & h_{33} & h_{34} \\ 0 & 0 & h_{43} & h_{44} \end{matrix} \right)\left( \begin{matrix} d_{1} \\ d_{2} \\ d_{3} \\ d_{4} \end{matrix} \right)=E\left( \begin{matrix} d_{1} \\ d_{2} \\ d_{3} \\ d_{4} \end{matrix} \right)\#\left( 3 \right) \end{aligned}$$

Finally, the energy of bonding state giving form:

$$\begin{aligned} E_{\pm}^{s}\left( \boldsymbol{k} \right)=\frac{1}{2}\left[ \left( h_{11}+h_{22} \right)\pm\sqrt{\left( h_{11}-h_{22} \right)^{2}+4h_{12}*h_{21}} \right]\#\left( 4 \right) \end{aligned}$$

The anti-bonding state giving form:

$$\begin{aligned} E_{\pm}^{a}\left( \boldsymbol{k} \right)=\frac{1}{2}\left[ \left( h_{11}+h_{22} \right)\pm\sqrt{\left( h_{33}-h_{44} \right)^{2}+4h_{34}*h_{43}} \right]\#\left( 5 \right) \end{aligned}$$

There is an angular variation of ARPES spectral weight, the matrix element effect, the intensity distribution of a given Bloch band depending on the momentum distribution of its associated Wannier orbital and the polarization. In this measurement, we used a right circularly polarized light. The matrix element is simulated as a *sin* function of angle *θ_q_* between ***q*** and *+k_x_* direction, similar to matrix element of monolayer graphene in a linear polarized light(*2*):

$$\begin{aligned} M_{CR}^{\pm}\left( \boldsymbol{k} \right)=\sin\left( \theta_{q}\left( \boldsymbol{k} \right)+\theta_{0}^{\pm} \right)\#\left( 6 \right) \end{aligned}$$

Here $\theta_{0}^{+},\theta_{0}^{-}$ are constants depending on electron state fitting by experiment. Considering the band spread depending on measurement energy resolution is a Gaussian distribution. We get the ARPES Intensity *I* at (*E*, ***k***) is:

$$\begin{aligned} I\left( E,\boldsymbol{k} \right)=\sum_{i=\pm,j=a,s} \frac{1}{\sigma\sqrt{2\pi}}\exp\left( -\frac{\left( E-E_{i}^{j}\left( \boldsymbol{k} \right) \right)^{2}}{2\sigma^{2}} \right)\cdot M_{CR}^{i}\left( \boldsymbol{k} \right)\#\left( 7 \right) \end{aligned}$$

Section S3: Absence of in-gap state with the presence of D-field.

There is no signature of additional in-gap state observed in ARPES spectra at different D-fields. In Fig. S2a, we presented an example of band dispersion of BLG with moiré superlattice taken at *V_bg_* = 15 V, and the corresponding energy distribution curve at *K*_BLG_ (green line in Fig. S2a) is presented in Fig. S2b. A clear V-shape gap (detailed explanation in section S13) is observed, indicative of the absence of additional in-gap state.


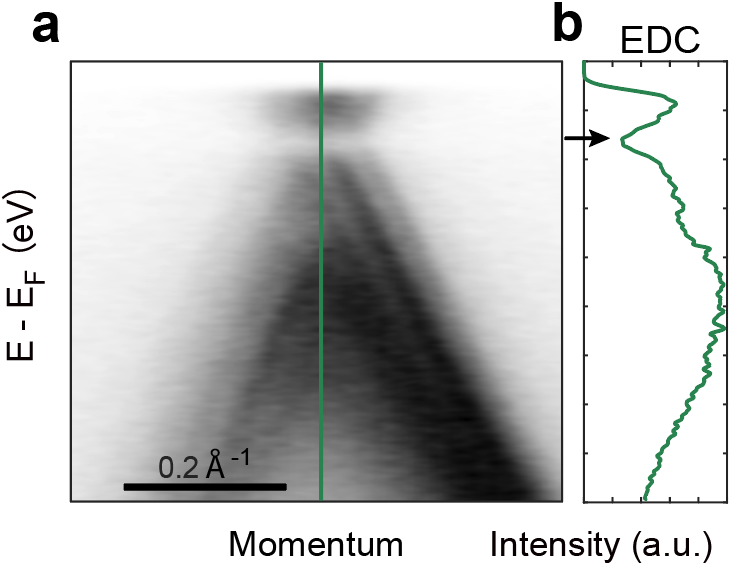


**Fig. S2: a.** ARPES band dispersion of BLG devices with moiré superlattice at *V_bg_* = 15 V. **b**. Energy distribution curve extracted from green line in **a**.

Section S4: Determination of twist angles of BLG/ hBN devices.

In an aligned BLG/hBN configuration, moiré replica of the original BLG bands is observed in ARPES. We use the simulated result, illustrated above in section S2, to fit the observed ARPES spectra and find the corresponding mini-BZ (illustrated in Fig. 2B of the main text). The twist angle between BLG and hBN can then be determined from the size of this mini-BZ.

For the case of unaligned BLG/hBN devices, there are no obvious moiré replicas. Alternatively, we use the angle between the K points of BLG and hBN to roughly estimate the twist angle. As illustrated in Fig. S3, the twisted angle in our device is extracted to be around 15°.


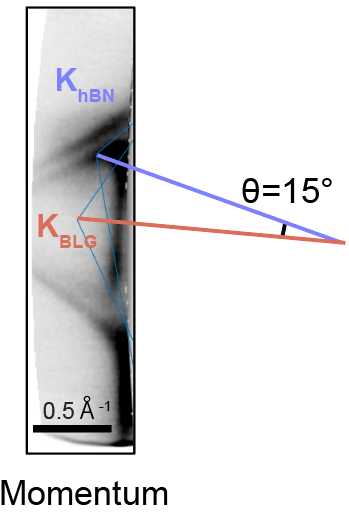


**Fig. S3.** Constant energy contours (-2 eV below *E_F_*) around K_BLG_ and K_hBN_, where the twisted angle can be roughly estimated between these K points.

Section S5: Calculation of the displacement field.

In a dual-gated device, with a stacking order of top gate-dielectric-BLG-dielectric-back gate, there are two displacement fields *D_t_* and *D_b_* generated in the top and bottom gate respectively (*3*, *4*). As a result, the doping level of the gated device can be estimated by treating the systems as two parallel plate capacitors:

$$\begin{aligned} n\propto\frac{Q_{t}-Q_{b}}{A}=\frac{C_{t}V_{t}}{A}-\frac{C_{b}V_{b}}{A}=\frac{\varepsilon_{t}V_{t}}{d_{t}}-\frac{\varepsilon_{b}V_{b}}{d_{b}}=D_{t}-D_{b}=\delta D \#\left( 8 \right) \end{aligned}$$

In addition, $\bar{D}=(D_{b}+D_{t})/2$ serves as the mean displacement field on BLG, which breaks the inversion symmetry of the bilayer graphene resulting in a bandgap at Dirac point (*3*, *4*). Therefore, with the application of both top and bottom gate voltages, we can individually control the displacement field $\bar{D}$ (keeping $\delta D$ constant) or the doping level $\delta D$ (keeping $\bar{D}$ constant).

Since ARPES is surface sensitive experiment and the sample has to be exposed to vacuum, we could only apply electric field with back gate and dielectric (hBN) from the bottom of the BLG (See Fig.1a, b in the main text) while grounding the top surface. In this situation, we assume there is no top gate voltage (and displacement field) on the top surface of the sample, e.g. $D_{t}=0$. With the application of only $D_{b}$, we always have $n\propto\delta D=-D_{b}, \bar{D}=D_{b}/2$. As a result, we lose the capability of tuning the doping level or the displacement field independently, as these two quantities change together with the back gate.

From our ARPES result, the change of the doping level manifests as the rigid shift of band positions towards higher binding energies and the band gap is observed at Dirac point resulted from the displacement field. The displacement field is calculated as:

$$\begin{aligned} \bar{D}=\frac{D_{b}}{2}=\frac{\varepsilon_{b}V_{b}}{{2d}_{b}}\#\left( 9 \right) \end{aligned}$$

where we use $\epsilon_{b}=\epsilon_{hBN} \approx2$ for hBN film (*5*), in the unit of $\epsilon_{0}=1$.

Section S6: Analysis of secondary Dirac point (SDP) gap.

The secondary Dirac points (SDPs) are areas where the moiré bands intersect (Fig. S4a), which is reported to open a gap around 25 meV in monolayer graphene with moiré superlattice (from alignment with hBN) (*6*–*9*). In the BLG with moiré superlattice, this impact of moiré potential is less pronounced, however still observable (*10*). Scanning tunneling spectroscopy (STS) measurement of BLG sample with moiré superlattice at different gating voltages is presented in Fig. S4b. A small dip is observed below the primary Dirac point gap (PDP) in the dI/dV curve, corresponding to the energy position (which does not change with gating) of the theoretically predicted SDP gap (Fig. S4c). The size of this SDP gap is estimated to be ~18 meV, consistent with the theoretical prediction (*10*). In our ARPES result, the SDP gap is expected to appear at where the main and moiré Dirac bands intersect (highlighted by the orange arrow in Fig. S4d), however its small size is beyond the limit of current energy resolution of NanoARPES experiment (see gating dependent result in Fig. S5).


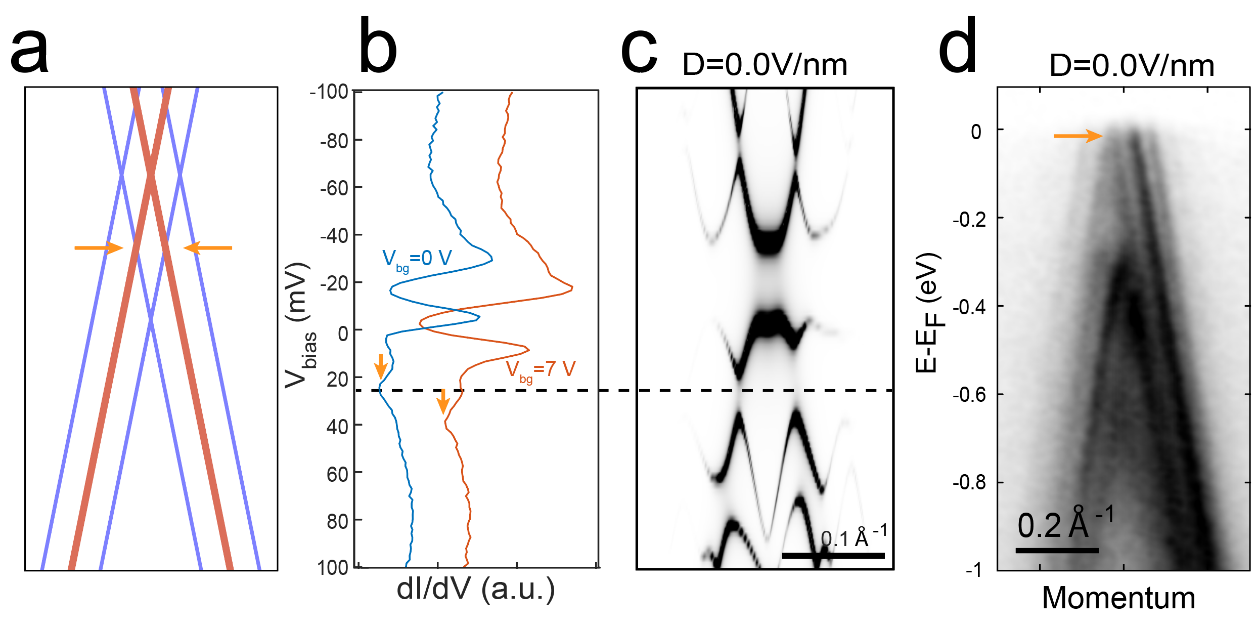


**Fig. S4 Electronic structure** of BLG with moiré superlattice **near E_F_. a.** Sketch of secondary Dirac point. **b**. Scanning tunneling spectroscopy (STS) of BLG with moiré superlattice at different gating voltage. **c.** Theoretical electronic structure at zero field. **d.** ARPES measurement at zero field.


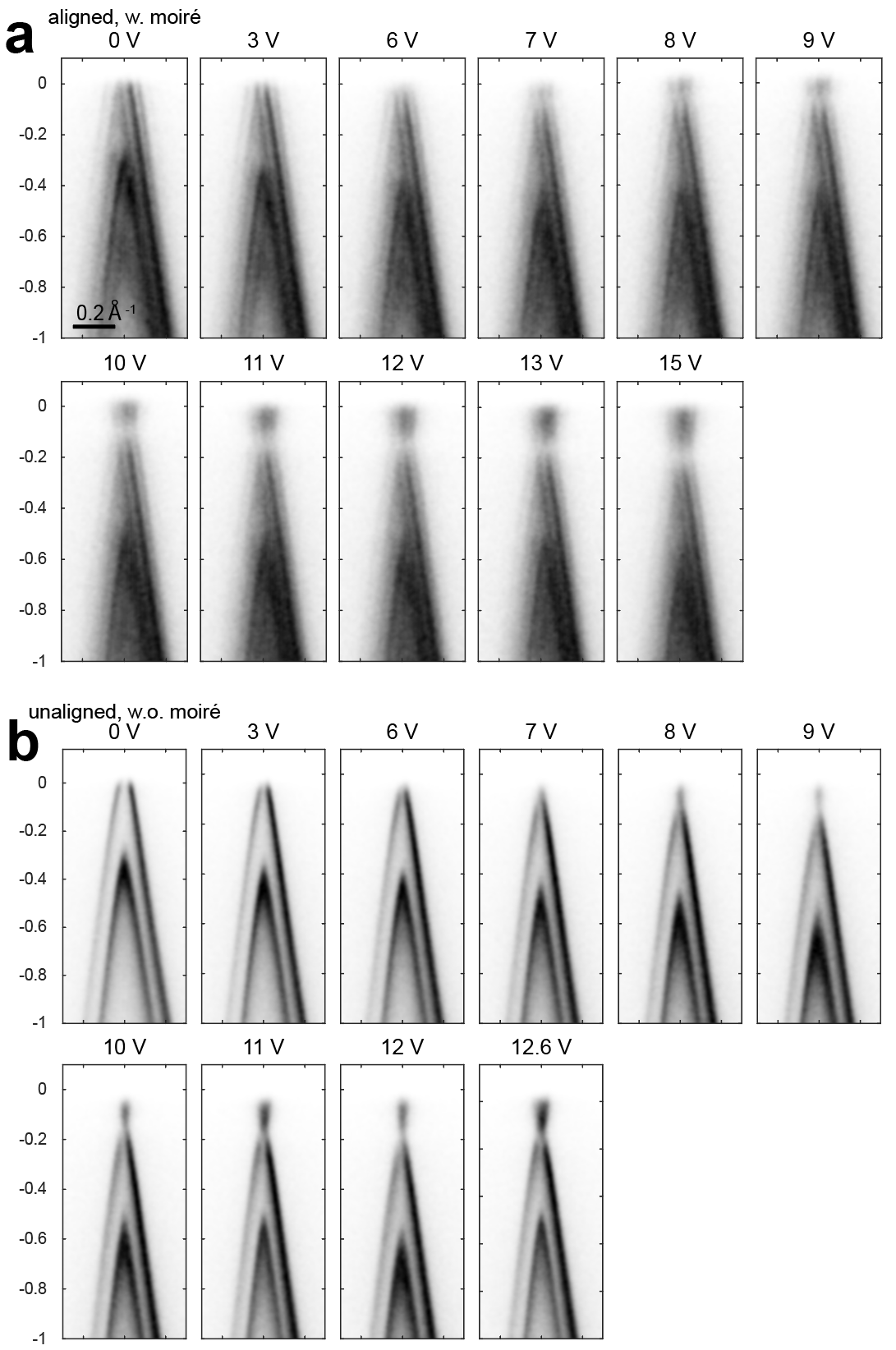


**Fig. S5.** Band dispersion of **a.** BLG with moiré superlattice and **b.** BLG without moiré superlattice at different gating voltage.

Section S7: Deviation of the ARPES extracted Δ_k_ from the theory predictions.

As presented in the Fig. 4D of the main text, the ARPES extracted Δ_k_ deviate slightly from the theoretical calculation, specifically at the high electric fields. A kink (decrease in slope) appears in the calculated bandgap. This feature can be attributed to interaction between the bandgaps of the primary Dirac point (PDP) and secondary Dirac point (SDP) (*11*).

The bandgap of the PDP increases linearly with the displacement field (Fig. 4C and (*11*)), whereas the bandgap of the SDP does not. Consequently, at a certain field, when the lower branch of the PDP intersects with the isolated flat band defined by the SDP gap, the overall bandgap is defined by the upper branch of the PDP and the flat band from the SDP, rather than PDP itself. This transition leads to the kink feature in the evolution of the calculated bandgap in the BLG with moiré superlattice system.

In the experiment result, however, as discussed previously in section S6, the SDP is not well observed, indicative of a relatively weaker influence from the moiré potential in this BLG/hBN configuration. Naturally, the predicted kink feature is also less prominent.

Section S8: Determination the thickness of hBN substrate.

We carried out AFM measurement to measure the thickness of the hBN substrate. As depicted in Fig. S6, the thickness is determined to be 19.0 nm in BLG device with moiré superlattice and 5.9 nm in BLG device without moiré superlattice, respectively.


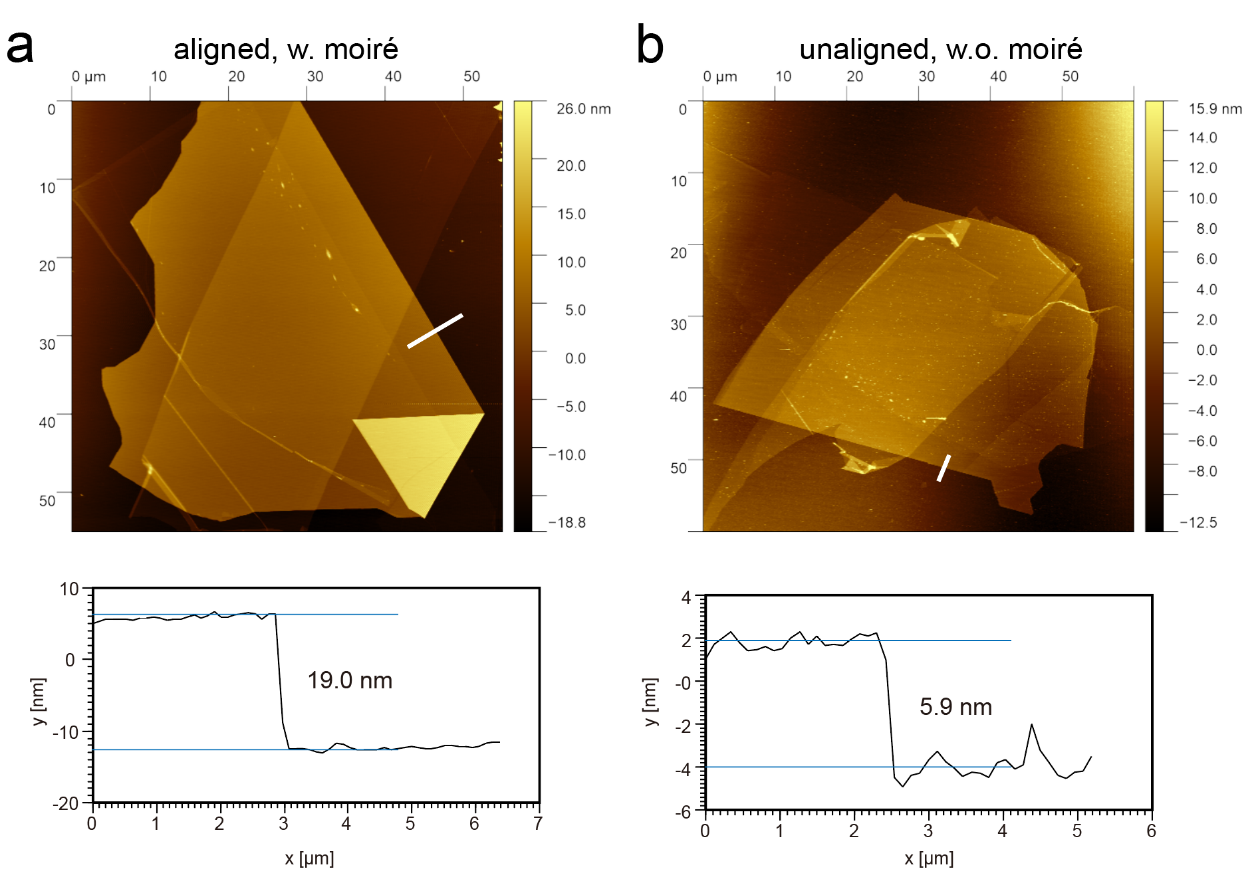


**Fig. S6. AFM hBN thickness measurements. a.** (upper) AFM mapping of BLG device with moiré superlattice. (lower) Height measurements of hBN at white solid line in upper Figure. **b.** Same but for BLG device without moiré superlattice.

Section S9: Homogeneity of the moiré lattice and moiré induced gap.

The electronic structure and the moiré induced gap can be greatly affect by lattice homogeneity. Therefore, we perform AFM measurements and NanoARPES experiments at different spatial locations, to evaluate homogeneity of the sample.

We capture Lateral Force Microscopy (LFM) real-space images at various locations on the BLG devices with moiré superlattice, as shown in Fig. S7. The average moiré length scale is ~ 13.5 nm, with no significant changes in shape and size over large areas (500 nm). This consistency suggests the absence of strain and twist variation, confirming the homogeneity of the moiré superlattice.

To further assess the uniformity of the electronic structure in BLG/hBN heterostructures, we conducted NanoARPES measurements at multiple spatial locations. As depicted in Fig. S8, we scrutinized the photoemission results from two BLG devices with moiré superlattice and one without moiré superlattice. The photoemission spectra at the K point revealed the distinctive electronic structure of BLG, along with replicas induced by the moiré lattice in the devices with moiré superlattice. These features remained consistent across different locations, confirming the uniformity of the electronic structure in our devices.

We also investigated the consistency of the moiré-induced band gap in the BLG devices with moiré superlattice using electrostatic gating at two different spatial locations under identical conditions. Fig. S9 illustrates the photoemission results at gate voltages of 0V, 8V, and 11V. The extracted band gap sizes were consistent across both locations, suggesting a uniform band gap influenced by the moiré lattice and the applied electric field.


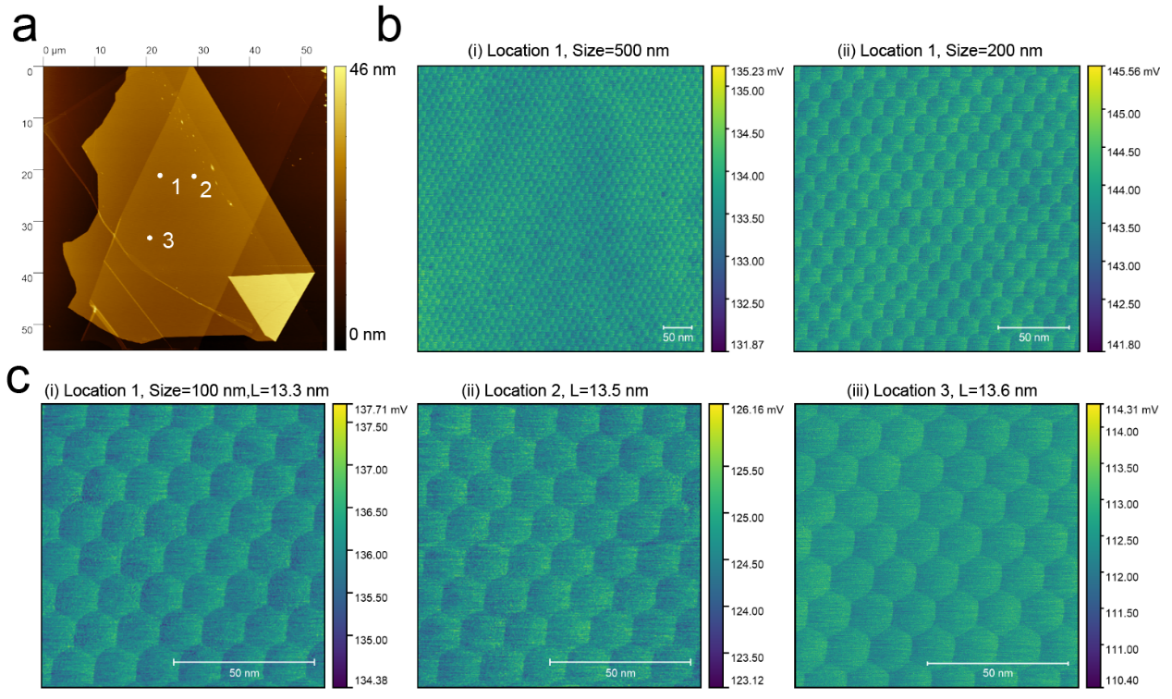


**Fig. S7. AFM characterization of the moiré lattice in BLG devices with moiré superlattice. a.** large scale AFM topography of sample. Labelled different measured locations. **b.** Moiré superlattice in different scale using Lateral Force Microscopy (LFM) at location 1. **c (i-iii)** Moiré superlattice at different locations labelled in a. The size is about 13.5 nm, consistent with the NanoARPES result 12 nm.


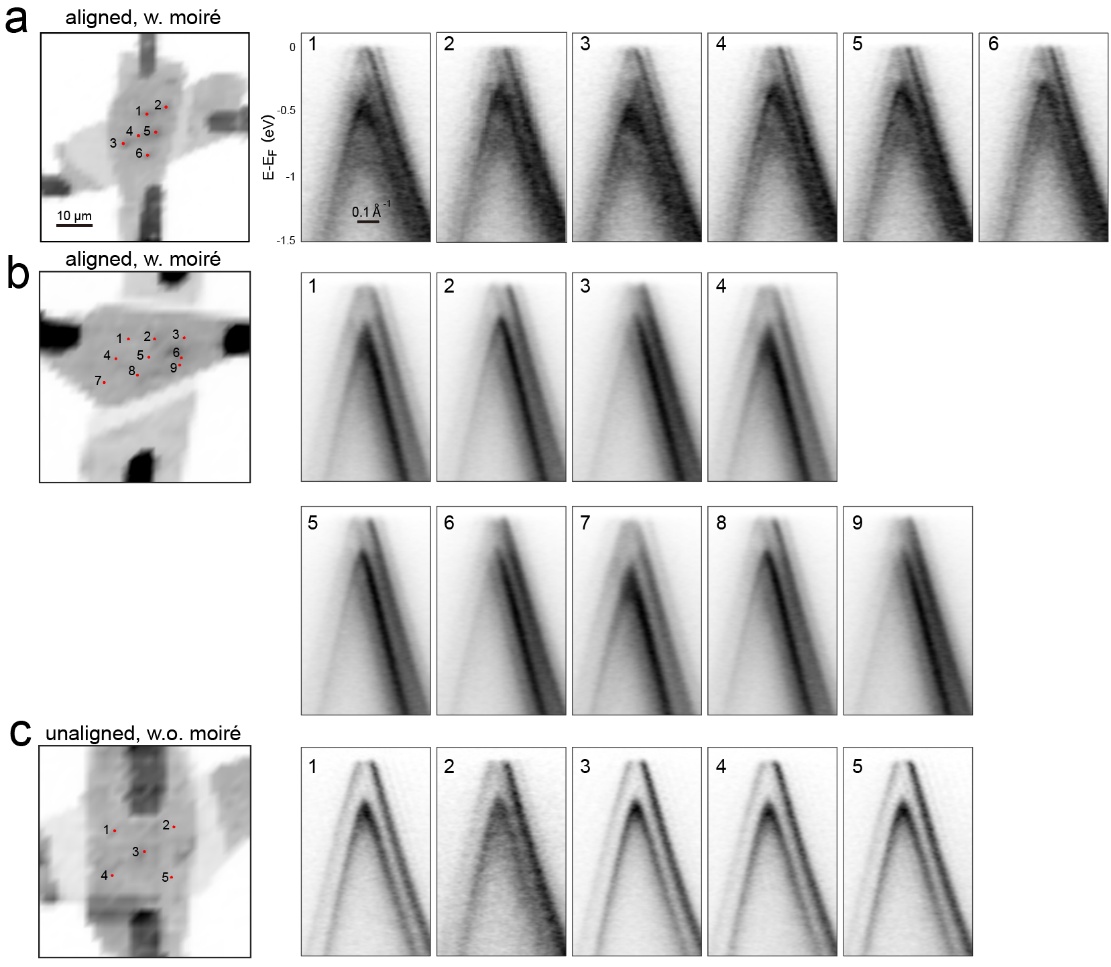


**Fig. S8.** **Measured band dispersion at different locations on BLG devices with/without moiré superlattice at zero gate voltage. a.** (left) NanoARPES real space intensity map for BLG devices with moiré superlattice. (right) Band dispersion across *K*_BLG_ at different locations labelled by red points in real space intensity map. **b.** Same as a, but for another BLG devices with moiré superlattice. **c.** Same as a but for BLG devices without moiré superlattice.


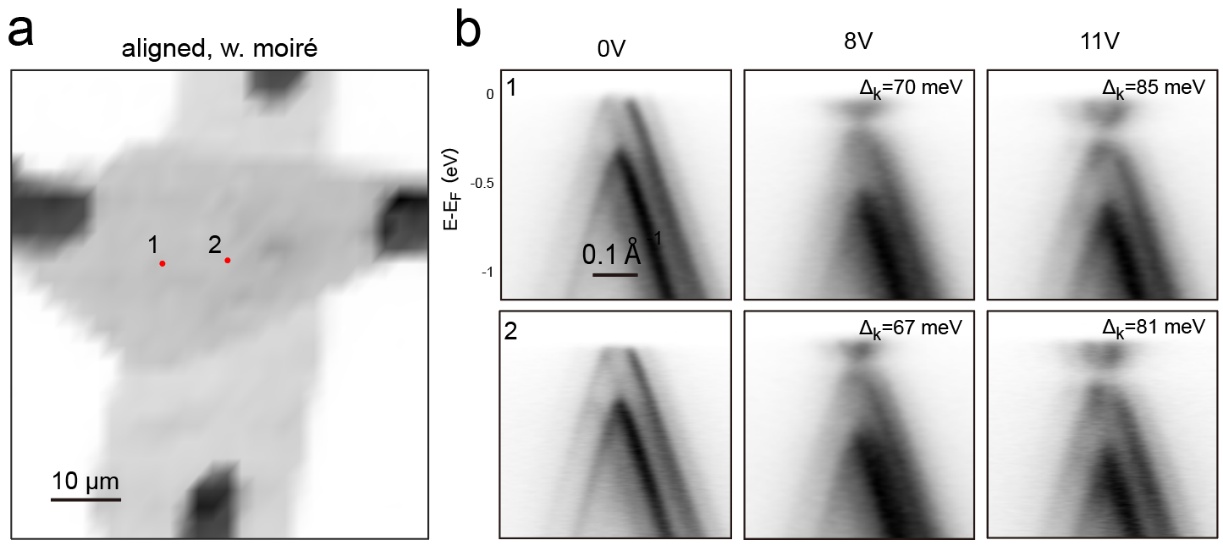


**Fig. S9. Measured band dispersion at different locations on BLG devices with moiré superlattice at finite gate voltages. a.** NanoARPES real space intensity map for BLG devices with moiré superlattice. **b.** Band dispersion across *K*_BLG_ at different locations labelled in a. Measured at gate voltage V_bg_ = 0V (1^st^ column), 8V (2^nd^ column), 11V (3^rd^ column), with labelled Δ_k_.

Section S10: Relaxation effects within the moiré lattice.

Structural relaxation within the moiré unit cell can lead to a reduction in the band gap at the Dirac point (*11*). In section S9, we discussed the spatial homogeneity of the devices and the repeatability of the observed band features. However, the AFM scanned image does not provide sufficient detail to address the effects of structural relaxation within the moiré unit cell. Therefore, to thoroughly investigate the electronic structure and the impact of structural relaxation, we conducted preliminary scanning tunneling microscope/spectroscopy (STM/S) measurements on BLG with moiré superlattice.

The moiré wavelength can be determined by topography mapping (Fig. S10b), approximately 13.5 nm, consistent with the AFM results (Fig. S10a). By analyzing the shapes of the AA stacked regions (bright triangles) and AB stacked regions (dark triangles), we observed that both regions are nearly equilateral triangles, suggesting minimal structural relaxation effects (refer to (*11*)).

Although a quantitative estimate of the structural relaxation (using the model from ref (*12*)) is beyond the scope of the manuscript, we evaluated its impact on the electronic structure through typical STS measurements at AA/AB’/AB sites (denoted as C/B/A in Fig. S10b, respectively). The STS measurements at these sites (Fig. S10c) revealed a consistent overall density of states near E_F_ across all locations. Specifically, the gap at the Dirac point, indicated in Fig. S10c, was estimated to be 18 meV at each site. This value aligns with the NanoARPES measurements and agrees with theoretical predictions of around 30 meV under unrelaxed conditions (see calculation results in Fig. 4C; relaxed conditions predict a typical gap size of less than 10 meV, as referenced in (*11*)). These results suggest the validity of our measurements and calculations, as well as the relatively minor role of structural relaxation in the BLG/hBN heterostructure.


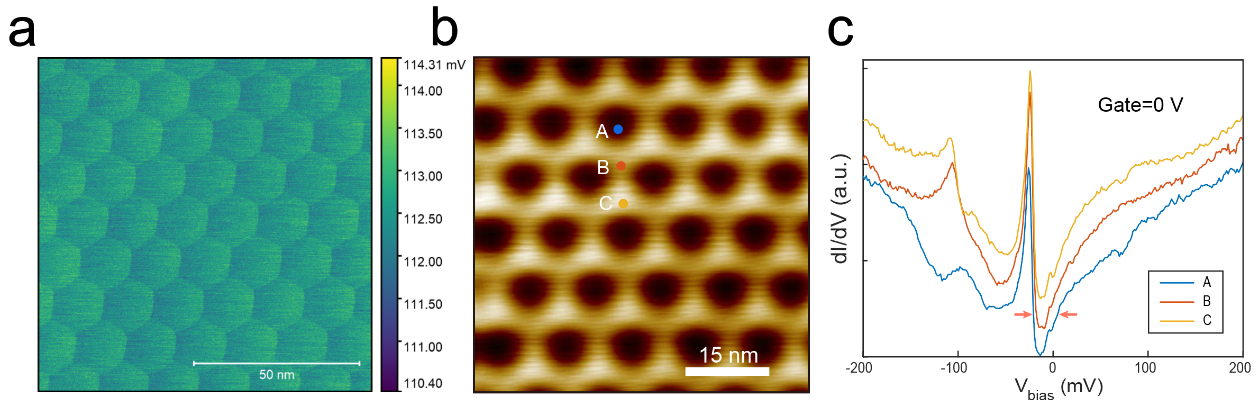


**Fig. S10. AFM/STM mapping on the moiré structure of BLG devices with moiré superlattice. a**. LFM mapping of device, showing the moiré superlattice. **b**. STM topography mapping, showing the detailed structure (AA, AB’ and AB sites) inside the moiré superlattice. **c**. dI/dV curves measured at three different sites labelled in **b**.

Section S11: Band gap size obtained through resistivity measurement.

To compare the band gap size obtained from different experimental methods, we carried out transport measurement on the same BLG devices with moiré superlattice. Resistivity (R_xx_) mapping with temperatures from 5K to 300K and gating voltage *V_bg_* from -0.25 to +0.25 V is illustrated in Fig S3a-b, and a logarithmical plot of R_xx_ with T^−1^ for three different gating voltages is presented in Fig. S11c.

At low temperatures, R_xx_ only weakly depends on 1/T, consistent with predictions from both variable range hopping (VRH) (*13*, *14*) and a combination of nearest-neighbor hopping and VRH (*4*). The low resistance values observed at low temperatures are reminiscent of those observed in disordered semiconductors where transport via impurity bands and hopping transport dominates. This notion is also in agreement with earlier studies (*15*) and measurements (*8*, *9*), which have shown that there is a large density of (localized) states in gapped BLG, resulting in low values of R_xx_.

At high temperatures T > 40 K, the dependence of R_xx_ on 1/T is well described by thermally activated transport $R_{xx}\propto exp(\Delta_{k}/2k_{B}T)$. We can extract Δ_k_ in the Arrhenius plot as shown in Fig S3c. In Fig S3d we calculated Δ_k_ at different V_bg_. It takes maximum at V_bg_ = 0.04V with Δ_k_ = 8.59 meV. This value is on the same order of magnitude but smaller than Δ_k_ ~ 20 meV extract from ARPES and Δ_k_ ~ 18 meV from STS (see Fig. S10c). This difference comes from the fact that ARPES/STS only probes the local electronic properties in a submicron/nanometer scale, while transport experiment measures the overall behavior of the sample. The transport result can be easily affected by the inhomogeneities of the sample, including impurity bands and hopping mentioned above, and low energy subgap edge state of BLG. Previous studies have reported sizable contribution from the edge state to the linear conductance of realistic devices (*16*), which is not currently accessible in ARPES measurement.


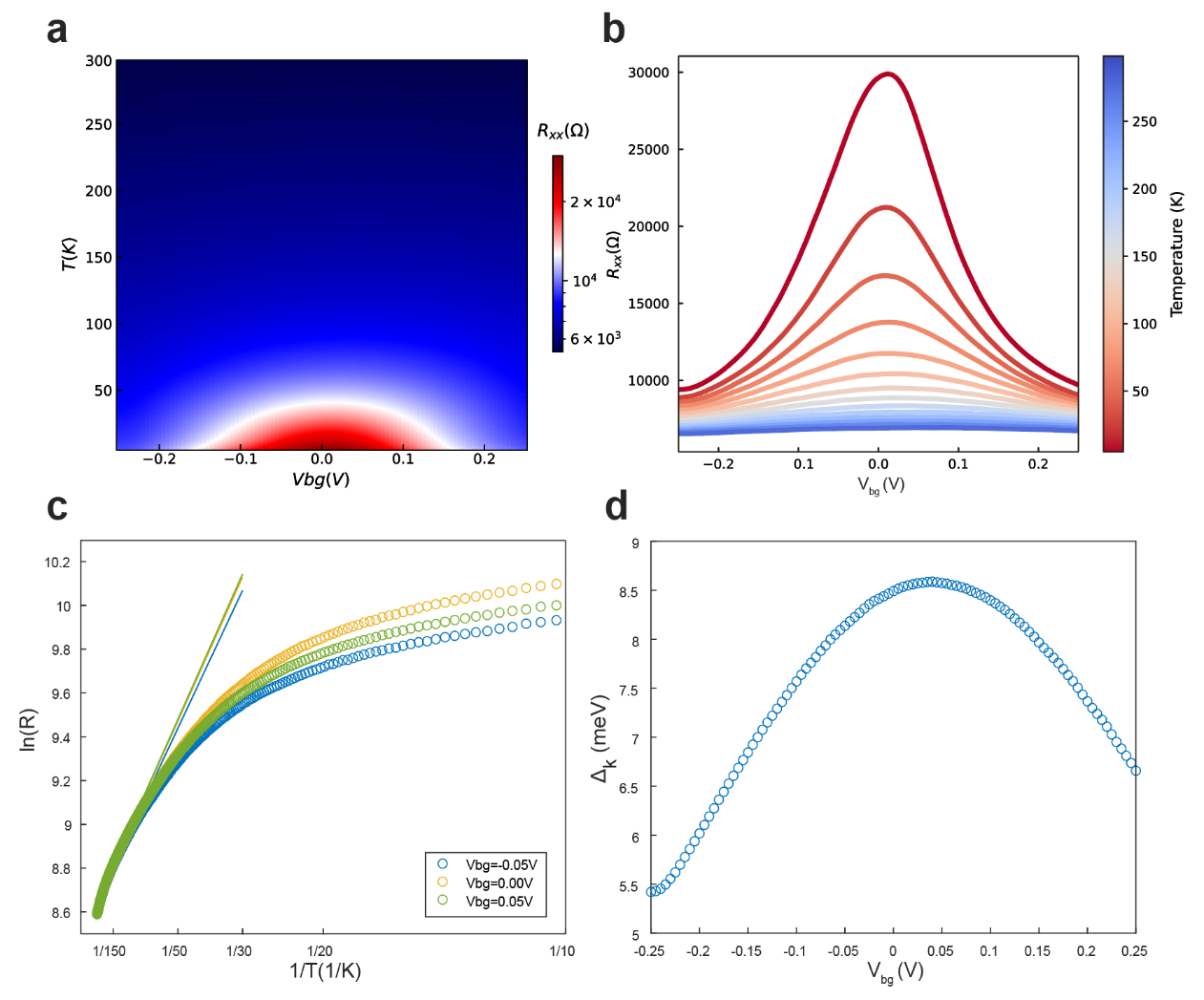
 **Fig. S11.** **Resistivity mapping experiment at varying temperatures on the BLG devices with moiré superlattice. a.** Color plot of R_xx_ as a function of back gate voltages and temperatures with V_sd_ = 50 μV. **b.** Line plot of a. R_xx_ takes maximum at V_bg_ ~ 0.04 V; **c.** Arrhenius plot showing ln(R_xx_) as a function of 1/T at three different V_bg_. **d**. Δ_k_ extracted from R_xx_ and T by thermally activated transport $R_{xx}\propto exp(\Delta_{k}/2k_{B}T)$. Δ_k_ takes maximum 8.59 meV at V_bg_ ~ 0.04 V.

Section S12: Exclusion of ferroelectricity in our device.

Interfacial ferroelectricity has been reported (*17*) in hBN/BLG/hBN system recently. To exclude the potential influence of similar ferroelectricity effect on the band gap, we further performed resistivity (R_xx_) measurement with forward/backward sweeping of the back gate voltage on the same sample measured in ARPES. As illustrated in Fig. S12, we observe no obvious signature of hysteretic behavior in the sweeping curves, indicative of the absence or nondetectable influence of the ferroelectricity in this BLG device with moiré superlattice.


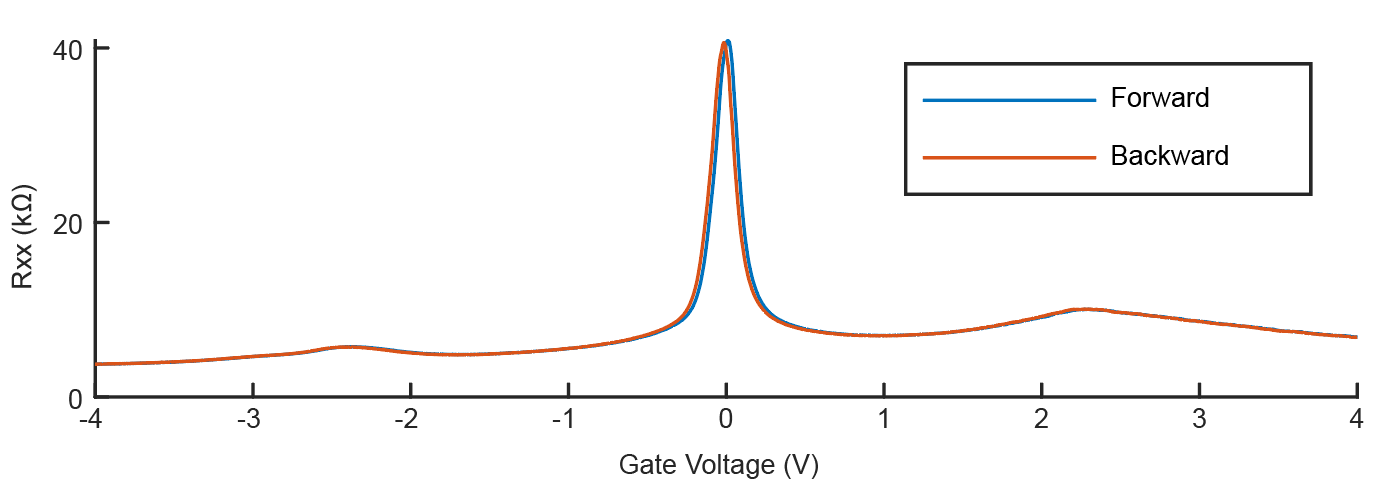


**Fig. S12. Longitudinal resistance of the BLG device with moiré superlattice measured by sweeping the back gate voltage**. **The measuring temperature was T=1.5K.**

Section S13: Extraction of Δ_k_ and the estimation of the measurement error.

To quantitatively distinguish the effects of moiré potential and D-field in BLG system, we extracted bandgap ∆*_k_* from NanoARPES spectra in Fig. 4. Below, we would explain step-by-step how we extract the band gap and estimate the error bar.

1. **The definition of measured band gap Δ_k_ .**
    When a clear quasiparticle peak defining the band dispersion is present, we can evaluate the gap size by identifying the peak position from the energy distribution curve across the band top and measuring the peak-to-peak energy spacing between two bands (see schematic in Fig. S13a). However, in cases where multiple adjacent bands are present, such as in BLG with moiré superlattice where main bands and moiré bands are close to each other, the multiple peaks can mix, making it difficult to identify the topmost/bottommost valence band (see schematic in Fig. S13b). Additionally, peak identification may not be accurate for determining the top/bottom of bands with a small effective mass, such as the Dirac bands in BLG devices with and without moiré superlattice. In such cases, the spectral weight is dominated by the spectrum close to, but not exactly at, the band top/bottom. In these situations, a common practice is to extract the band top/bottom position from the half-maximum point on the topmost/bottommost "leading edge" of the energy distribution curve of the ARPES spectrum (see yellow dashed line in Fig. S13b). This method is used to evaluate multiple types of gaps (band gap, superconducting gap, and charge density wave gap) in ARPES measurements (ref (*18*) and (*19*) for the gap analysis in graphene based system). This approach is physically valid as the photoemission spectral function directly reflects the momentum-dependent density of states, making it reasonable to extract a gap from the energy position where the density of states drops to half.

There is also a density of state within the gap observed in Fig. 2E and 3E, which is coming from 1) the secondary incoherent electrons which increases gradually as a function of binding energy and 2) spectrum broadening effect due to the phonon and impurity scattering of electrons (fermi liquid behavior). The ARPES instrumentation energy/momentum resolution further broadens the peaks. With these effects, ARPES never detects an “clear gap” with no in-gap detector count. A simulation in Fig. S13d shows this in-gap intensity with a gapped Dirac band spectrum broadened to width similar to the experiment observation, justifying the dip we observed are from the actual bandgap. Instead, a clear dip may be observed (but not always) from STS measurement (see Fig. S10c). The same moiré gap size measured from STS and ARPES extrapolation justifies our quantitative measurement of the bandgap in BLG/hBN system.

1. **Estimate of the error bar.** Given the methods we use to extract the bandgap, there would be two types of uncertainties in estimating the bandgap size:
   - 1. Sample independent measurement uncertainty. This part comes from the ARPES instrument, including the beam monochromaticity resolution (~10 meV) and electron energy analyzer resolution (~15 meV and ~0.2º). The total energy resolution could be estimated by fitting the photoemission spectrum near E_F_ at temperature T using the function $\left( A\left( E, k \right)*\frac{1}{\exp\left( \frac{E-E_{F}}{k_{B}T} \right)+1} \right)\bigotimes\Delta_{1}\left( E \right)$, where the intrinsic spectrum times the Fermi Dirac function at T and convolves with the instrument energy resolution $\Delta_{1}\left( E \right)$. For the two samples, the spectrum fitting gives the total instrument resolution $\Delta_{1}\left( E \right)$ to be 18.9 meV (Fig. S13c).
     2. Sample-dependent uncertainty $\Delta_{2}\left( E \right)$ arising from the variation of broadness of the quasiparticle spectral function. According to Fermi liquid theory, the broadness of the peak is proportional to the imaginary part of the quasiparticle's self-energy and inversely proportional to the quasiparticle lifetime. The broadness is dependent on the sample, temperature, and carrier concentration, as variations in impurity levels, phonon scattering rates, and electron binding energy affect the quasiparticle lifetime.

To estimate the spectrum broadness, we use a 2D Gaussian function $G\left( E,k \right)=\exp\left( \frac{k^{2}}{2\sigma_{k}^{2}}+\frac{E^{2}}{2\sigma_{E}^{2}} \right)$, which describes the total energy and momentum uncertainties. We estimate $G\left( E,k \right)$ by convolving it with a linear band having the same Fermi velocity as the Dirac bands observed from ARPES measurements and then matching both the momentum and energy widths of the resulting spectrum with the experimentally observed ones (see Fig. S14).

Since the broadness changes from sample to sample, it’s reasonable to take part of it as uncertainty for evaluation of the gap (since it could not reduce to 0). Therefore, sample-dependent uncertainty $\Delta_{2}\left( E \right)$is estimated as the half the$\sigma_{E}$of $G\left( E,k \right)$, and ~ 20 meV. We found $\Delta_{2}\left( E \right)$ to be larger than $\Delta_{1}\left( E \right)$ because the instrument resolution has already been taken into account when measuring $\Delta_{2}\left( E \right)$_._

After taking these accounts into consideration, we take the sample and gate dependent $\Delta_{2}\left( E \right)$ as the total uncertainty of the extracted band gap sizes**.**

We plot several typical experimental spectra and fitted spectrum with extracted $\sigma_{E}$and $\sigma_{k}$labelled in Fig. S14.

We believe the above discussions and revisions would provide a more systematic and accurate description of our data processing procedure and quantitative analysis of the bandgap.


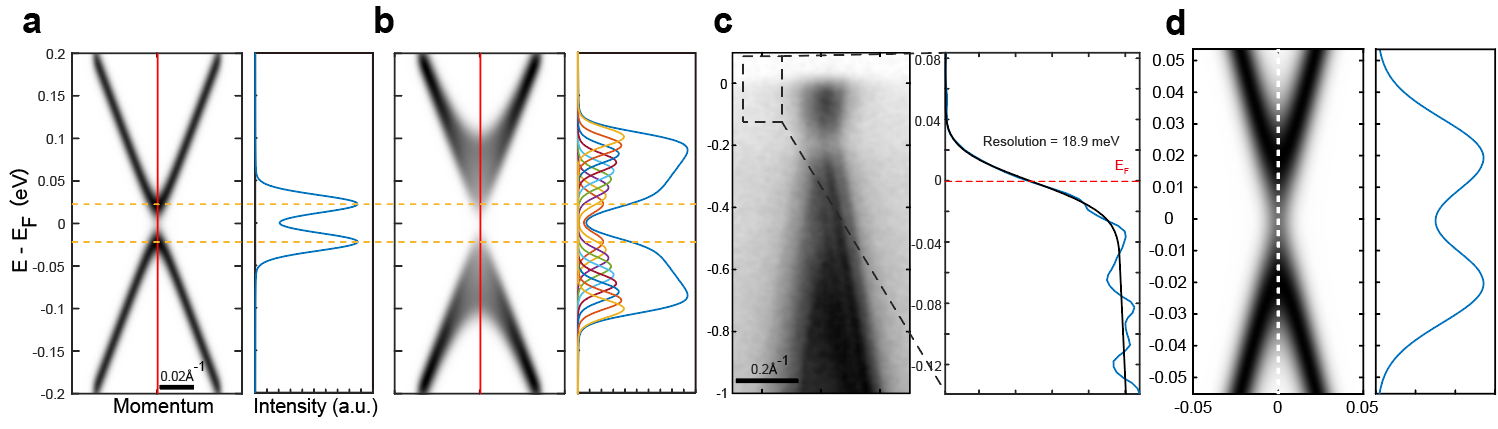


**Fig. S13. Schematic of gap analysis and fitting of system energy resolution a.** Simulation of a gapped Dirac cone with 40 meV gap with spectrum broadening, and its energy distribution curve. **b.** Same as **a** but containing several adjacent bands with different band top/bottoms. **c**. Resolution fitting from BLG with moiré superlattice data at V_bg_ = 15 V. **e**. Schematic simulation of ARPES spectrum with fully gapped Dirac cone band dispersion, with spectrum broadening, and its corresponding energy distribution curve across the Dirac point.


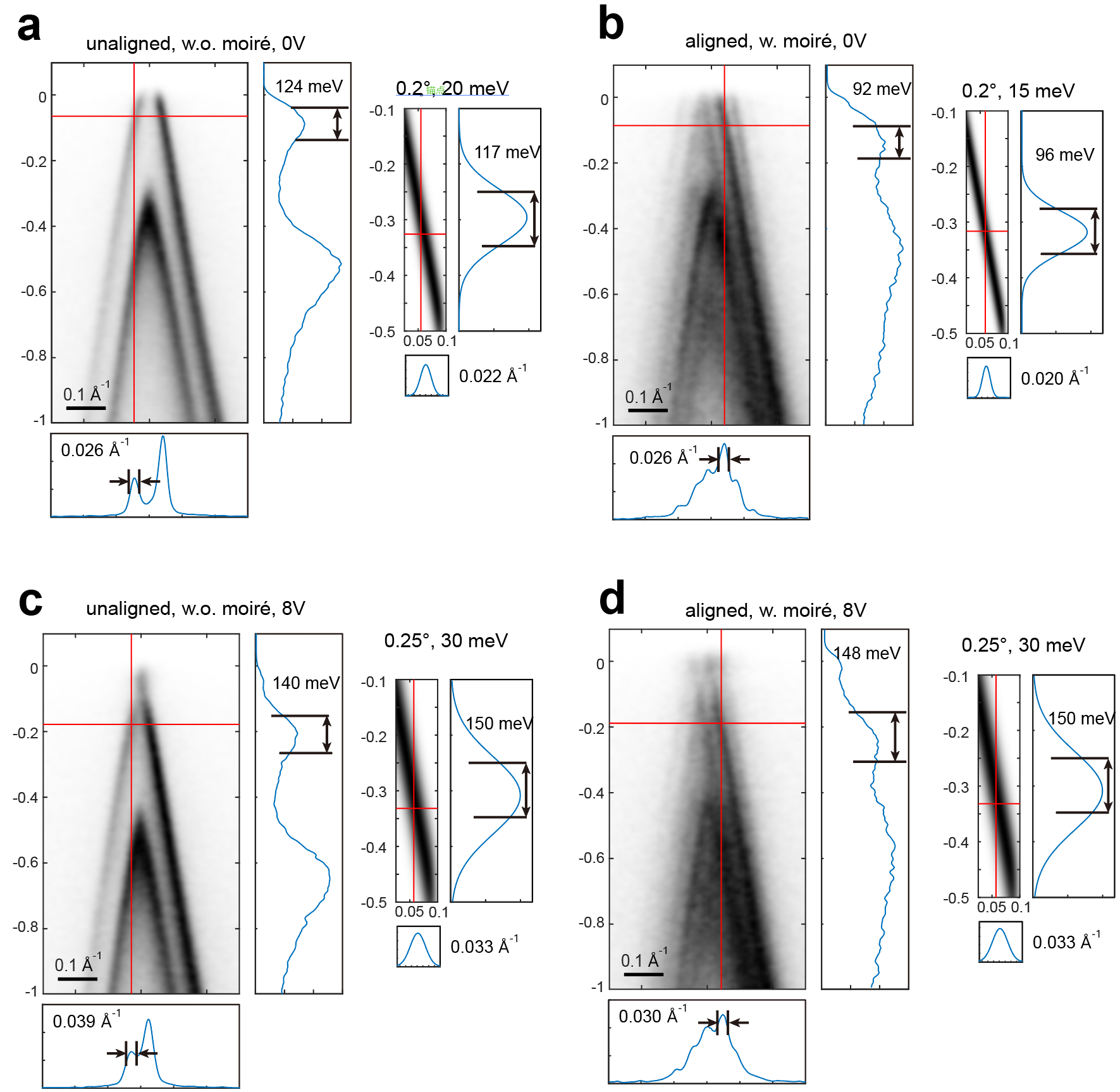


**Fig. S14. Simulation of a Dirac band dispersion with spectrum broadening, and energy distribution curve at K point.** For each spectrum, a linear dispersion with same velocity as the Dirac band is convolved with $G\left( E,k \right)=\exp\left( \frac{k^{2}}{2\sigma_{k}^{2}}+\frac{E^{2}}{2\sigma_{E}^{2}} \right)$, The evaluated $\frac{\sigma_{E}}{2},\frac{\sigma_{k}}{2}$is labelled. The fitted energy distribution curve and momentum distribution curve is compared with the measured experiment spectrum to match the width in energy and momentum.

References

1. C. P. Chang, Analytic model of energy spectrum and absorption spectra of bilayer graphene. *Journal of Applied Physics* **111**, 103714 (2012).

2. C. Hwang, C.-H. Park, D. A. Siegel, A. V. Fedorov, S. G. Louie, A. Lanzara, Direct measurement of quantum phases in graphene via photoemission spectroscopy. *Physical Review B* **84**, 125422 (2011).

3. Y. Zhang, T.-T. Tang, C. Girit, Z. Hao, M. C. Martin, A. Zettl, M. F. Crommie, Y. R. Shen, F. Wang, Direct observation of a widely tunable bandgap in bilayer graphene. *Nature* **459**, 820–823 (2009).

4. M. Sui, G. Chen, L. Ma, W.-Y. Shan, D. Tian, K. Watanabe, T. Taniguchi, X. Jin, W. Yao, D. Xiao, Y. Zhang, Gate-tunable topological valley transport in bilayer graphene. *Nature Phys* **11**, 1027–1031 (2015).

5. S. Jung, N. Myoung, J. Park, T. Y. Jeong, H. Kim, K. Watanabe, T. Taniguchi, D. H. Ha, C. Hwang, H. C. Park, Direct Probing of the Electronic Structures of Single-Layer and Bilayer Graphene with a Hexagonal Boron Nitride Tunneling Barrier. *Nano Lett.* **17**, 206–213 (2017).

6. E. Wang, X. Lu, S. Ding, W. Yao, M. Yan, G. Wan, K. Deng, S. Wang, G. Chen, L. Ma, J. Jung, A. V. Fedorov, Y. Zhang, G. Zhang, S. Zhou, Gaps induced by inversion symmetry breaking and second-generation Dirac cones in graphene/hexagonal boron nitride. *Nature Phys* **12**, 1111–1115 (2016).

7. M. Yankowitz, J. Xue, D. Cormode, J. D. Sanchez-Yamagishi, K. Watanabe, T. Taniguchi, P. Jarillo-Herrero, P. Jacquod, B. J. LeRoy, Emergence of superlattice Dirac points in graphene on hexagonal boron nitride. *Nature Phys* **8**, 382–386 (2012).

8. B. Hunt, J. D. Sanchez-Yamagishi, A. F. Young, M. Yankowitz, B. J. LeRoy, K. Watanabe, T. Taniguchi, P. Moon, M. Koshino, P. Jarillo-Herrero, R. C. Ashoori, Massive Dirac Fermions and Hofstadter Butterfly in a van der Waals Heterostructure. *Science* **340**, 1427–1430 (2013).

9. R. Ribeiro-Palau, C. Zhang, K. Watanabe, T. Taniguchi, J. Hone, C. R. Dean, Twistable electronics with dynamically rotatable heterostructures. *Science* **361**, 690–693 (2018).

10. M. Kuiri, S. K. Srivastav, S. Ray, K. Watanabe, T. Taniguchi, T. Das, A. Das, Enhanced electron-phonon coupling in doubly aligned hexagonal boron nitride bilayer graphene heterostructure. *Phys. Rev. B* **103**, 115419 (2021).

11. R. Smeyers, M. V. Milošević, L. Covaci, Strong gate-tunability of flat bands in bilayer graphene due to moiré encapsulation between hBN monolayers. *Nanoscale* **15**, 4561–4569 (2023).

12. Z. Bi, N. F. Q. Yuan, L. Fu, Designing flat bands by strain. *Phys. Rev. B* **100**, 35448 (2019).

13. L. Jing, J. Velasco Jr., P. Kratz, G. Liu, W. Bao, M. Bockrath, C. N. Lau, Quantum Transport and Field-Induced Insulating States in Bilayer Graphene pnp Junctions. *Nano Lett.* **10**, 4000–4004 (2010).

14. J. Yan, M. S. Fuhrer, Charge Transport in Dual Gated Bilayer Graphene with Corbino Geometry. *Nano Lett.* **10**, 4521–4525 (2010).

15. H. Miyazaki, K. Tsukagoshi, A. Kanda, M. Otani, S. Okada, Influence of Disorder on Conductance in Bilayer Graphene under Perpendicular Electric Field. *Nano Lett.* **10**, 3888–3892 (2010).

16. J. Li, I. Martin, M. Büttiker, A. F. Morpurgo, Topological origin of subgap conductance in insulating bilayer graphene. *Nature Phys* **7**, 38–42 (2011).

17. Z. Zheng, Q. Ma, Z. Bi, S. De La Barrera, M.-H. Liu, N. Mao, Y. Zhang, N. Kiper, K. Watanabe, T. Taniguchi, J. Kong, W. A. Tisdale, R. Ashoori, N. Gedik, L. Fu, S.-Y. Xu, P. Jarillo-Herrero, Unconventional ferroelectricity in moiré heterostructures. *Nature* **588**, 71–76 (2020).

18. S. Y. Zhou, D. A. Siegel, A. V. Fedorov, A. Lanzara, Metal to insulator transition in epitaxial graphene induced by molecular doping. *Phys. Rev. Lett.* **101**, 86402 (2008).

19. H. Matsui, K. Terashima, T. Sato, T. Takahashi, M. Fujita, K. Yamada, Direct observation of a nonmonotonic dx2-y2-wave superconducting gap in the electron-doped high-Tc superconductor Pr0.89LaCe0.11CuO4. *Phys. Rev. Lett.* **95**, 17003 (2005).
